# Macrophage Migration Inhibitory Factor (MIF) Is Essential for Type 2 Effector Cell Immunity to an Intestinal Helminth Parasite

**DOI:** 10.3389/fimmu.2019.02375

**Published:** 2019-10-24

**Authors:** Kara J. Filbey, Fumi Varyani, Yvonne Harcus, James P. Hewitson, Danielle J. Smyth, Henry J. McSorley, Alasdair Ivens, Susanne Nylén, Martin Rottenberg, Stephan Löser, Rick M. Maizels

**Affiliations:** ^1^Institute of Immunology and Infection Research, University of Edinburgh, Edinburgh, United Kingdom; ^2^Wellcome Centre for Integrative Parasitology, Institute of Infection, Immunity and Inflammation, University of Glasgow, Glasgow, United Kingdom; ^3^Centre for Inflammation Research, University of Edinburgh, Edinburgh, United Kingdom; ^4^Department of Microbiology, Tumor and Cell Biology, Karolinska Institute, Stockholm, Sweden

**Keywords:** arginase, *Heligmosmoides polygyrus*, helminths, macrophage, eosinophil, innate immunity

## Abstract

Immunity to intestinal helminths is known to require both innate and adaptive components of the immune system activated along the Type 2 IL-4R/STAT6-dependent pathway. We have found that macrophage migration inhibitory factor (MIF) is essential for the development of effective immunity to the intestinal helminth *Heligmosomoides polygyrus*, even following vaccination which induces sterile immunity in wild-type mice. A chemical inhibitor of MIF, 4-IPP, was similarly found to compromise anti-parasite immunity. Cellular analyses found that the adaptive arm of the immune response, including IgG1 antibody responses and Th2-derived cytokines, was intact and that Foxp3^+^ T regulatory cell responses were unaltered in the absence of MIF. However, MIF was found to be an essential cytokine for innate cells, with ablated eosinophilia and ILC2 responses, and delayed recruitment and activation of macrophages to the M2 phenotype (expressing Arginase 1, Chil3, and RELM-α) upon infection of MIF-deficient mice; a macrophage deficit was also seen in wild-type BALB/c mice exposed to 4-IPP. Gene expression analysis of intestinal and lymph node tissues from MIF-deficient and -sufficient infected mice indicated significantly reduced levels of *Arl2bp*, encoding a factor involved in nuclear localization of STAT3. We further found that STAT3-deficient macrophages expressed less Arginase-1, and that mice lacking STAT3 in the myeloid compartment (LysM^Cre^xSTAT3^fl/fl^) were unable to reject a secondary infection with *H. polygyrus*. We thus conclude that in the context of a Type 2 infection, MIF plays a critical role in polarizing macrophages into the protective alternatively-activated phenotype, and that STAT3 signaling may make a previously unrecognized contribution to immunity to helminths.

## Introduction

Intestinal helminths constitute the most prevalent group of parasites in the human population today, with around 1.5 billion people infected throughout the tropical and sub-tropical zones of the globe (1, 2). While drugs are available that temporarily clear intestinal parasites, therapy does not confer immunity to re-infection. Strategies aiming to boost the immune system through vaccination are constrained by a lack of understanding of basic mechanisms of resistance to infection, including the relative roles of innate and adaptive immunity in expelling parasites (3). Thus, while CD4^+^ T cells are essential drivers of anti-helminth immunity, parasite expulsion requires activation of innate effector cell populations (4, 5). In the case of the mouse model parasite *Heligmosomoides polygyrus*, the most critical effector is likely to be the alternatively activated (M2) macrophage induced through T-cell derived IL-4/-13 (6, 7). Signaling through the IL-4Rα subunit is essential for M2 activation, and is known to require STAT6 activation and nuclear translocation (8, 9). However, the role of other STAT factors in immunity to helminths has been little explored (10).

We now identify a key player in immunity to *H. polygyrus* to be macrophage migration inhibitory factor (MIF), as mice genetically deficient in this protein, or exposed to pharmacological inhibitors of MIF, are unable to expel intestinal worms normally. Although MIF has been classically associated with type 1 inflammation during microbial exposure and sepsis (11, 12), more recent studies have also identified a role for this molecule in development of Th2 responsiveness to allergens (13, 14). It has also been reported that antibody-mediated neutralization of MIF *in vivo* increases the burden of *Schistosoma japonicum* worms in the tissues of infected mice (15). In addition, we and others have found that M2 activation of macrophages by IL-4 is amplified in the presence of MIF (16, 17). We therefore decided to test the role of MIF in chronic infection with *H. polygyrus*.

As described below, MIF-deficient animals show delayed infiltration and activation across a broad range of innate immune cell populations, including macrophages, type 2 innate lymphoid cells (ILC2s) and eosinophils. Gene array analyses of infected tissues pointed to a relatively circumscribed shift in expression profile which included sharply reduced levels of *Arl2bp*, a promoter of STAT3 function. Mice lacking STAT3 in their myeloid compartment were found to phenocopy the MIF-deficient mice in failing to reject a challenge infection of *H. polygyrus*. Hence, MIF is an essential mediator in the activation of the innate compartment for immunological clearance of parasitic helminths from the gastrointestinal tract, in a manner dependent at least in part upon signaling through STAT3.

## Materials and Methods

### Mice and Parasites

BALB/c and MIF-deficient mice on the BALB/c background (18) were bred in-house and housed in individually-ventilated cages (IVCs) according to UK Home Office guidelines. Mice on the C57BL/6 background expressing Cre recombinase under the LysM promoter (19), and carrying flanking loxP (flox) sites either side of the *Stat3* locus, were bred as described previously (20).

Infections employed 200 L3 larvae of *H. polygyrus* in 200 μl water by oral gavage. Parasite lifecycles and collection of excretory-secretory (HES) products were conducted as previously described in CBAxC57BL/6 F1 mice (21). Granuloma and adult worm counts were conducted after small intestines were removed and sliced longitudinally. Three to four fecal pellets were weighed and dissolved in 2 ml dH_2_0; 2 ml of saturated salt solution (400 g NaCl in 1 L dH_2_0) was then added and eggs enumerated using a McMaster egg counting chamber. Egg counts are represented as eggs/g fecal material. For secondary infections, mice were cleared of worms after infection with *H. polygyrus* with pyrantel embonate Strongid-P paste (Elanco Animal Health), given in 2 doses of 2.5 mg dissolved in 200 μl dH_2_0 given on days 28 and 29 by oral gavage. After 2 weeks, mice were re-infected with 200 L3 larvae.

### *In vivo* Administrations

One mg of MIF inhibitor, 4-IPP (Tocris Bioscience #3249) (22) dissolved in DMSO, or DMSO alone, was administered intraperitoneally (i.p.) in 50 μl every other day, during *H. polygyrus* infection [adapted from (17)]. Fifty ng of recombinant MIF (R&D) in 50 μl PBS, or PBS alone, was administered i.p. every other day, during *H. polygyrus* infection. rIL-33 (R&D) was administered intranasally (200 ng in 50 μl PBS) to sedated mice on days 0, 1 and 2, and lung tissue taken at day 3 for analysis. *Alternaria alternata* antigen (Greer) was administered intranasally (10 μg in 50 μl PBS) to sedated mice. BALF was harvested 1 h later (adapted from (23). For vaccination, mice were immunized with 5 μg of HES i.p. in alum adjuvant, and boosted on days 28 and 35 with 1 μg in alum before challenge with *H. polygyrus* at day 42 (24).

### Cell Isolation and Culture

Mesenteric lymph node (MLN) cell suspensions were prepared directly by passage through 70 μm nylon filters (BD) and placed in RPMI1640 (Gibco) containing 10% FCS, 1% PenStrep (Gibco) and 1% L-glutamine (Gibco) (complete RPMI). Cells were restimulated for 72 h at 37°C with either media alone or HES at a final concentration of 1 μg/ml with 1 × 10^6^ cells, in triplicate. Peritoneal exudate cells were collected by washing the peritoneal cavity with 2 × 5 ml RPMI1640 using a 23 gauge needle. Red blood cells were removed by adding 3 ml red blood cell (RBC) lysis buffer (Sigma) for 4 min, and washing with complete RPMI. Peritoneal lavage used for ELISA analysis consisted of the supernatant from the first 5 ml wash following centrifugation to pellet cells. Bronchoalveloar lavage was collected by washing the lungs with 1 ml ice-cold PBS. Lung tissue was digested in HBSS (Gibco) supplemented with 4 U/ml Liberase TL (Roche) and 160 U/ml DNAse 1 (Sigma). Tissue was incubated at 37°C for 25 min, passed through 70 μm nylon filters (BD) and RBC-lysed before cells were used for flow cytometric analysis.

### Flow Cytometry

Cells were stained in 96-well round-bottomed plates. Prior to antibody staining, cells were washed in PBS and stained with LIVE/DEAD Fixable Blue (Invitrogen) at a 1/1000 dilution in 100 μl PBS for 20 min at 4°C. Then, Fc receptors were blocked in 50 μl of FACS buffer containing 100 μg/ml of naïve rat IgG (Sigma) for 20 min at 4°C. Samples were then surface stained for 20 min in 20 μl of FACS buffer containing a combination of the antibodies detailed below. Lineage markers for ILC2 negative gating: CD3 (Biolegend 17A2), CD4 (Biolegend RM4-5), CD8α (Biolegend 53-6.7), CD19 (Biolegend 6D5) CD49b (eBioscience DX5), Gr1 (Biolegend RB6-8C5), CD11c (Biolegend N418); F4/80 (Biolegend BM8), CD11b (Biolegend M1/70), SiglecF (BD E50-2440); ICOS staining for ILC2 used BioLegend C398.4A. To measure intracellular IL-5, cells were first stimulated for 4 h at 37°C in the presence of PMA (50 ng/ml), Ionomycin (1 μg/ml), and Brefeldin A (10 μg/ml) (all from Sigma). For pSTAT6 staining, clone pY641 conjugated to AF647 (BD Cat No 558242) was used. Following surface staining, cells were permeabilised for 30 min at 4°C in Cytofix/Cytoperm solution (BD), and then washed twice in 200 μl of Perm/Wash (BD). ILC2s were stained for intracellular cytokine expression in Perm/Wash (BD) using anti-IL-5 (eBioscience TRFK5). For Foxp3 (eBioscience, FJK-16s), Arginase-1 (R&D Systems IC5868P), RELM-α [R&D Systems 226033, labeled with AF647 (Invitrogen)] and Chil3 [R&D biotinylated goat anti-mouse combined with Streptavidin PeCy7 (Biolegend)], samples were stained for surface markers after which cells were permeabilised for 12 h at 4°C in Fix/Perm solution (eBioscience Foxp3 staining set), and then washed twice in 200 μl of Perm/Wash (eBioscience Foxp3 staining set). After staining, cells were washed twice in 200 μl of FACS buffer before acquisition on the LSR II or Canto flow cytometers (BD Bioscience) and subsequently analyzed using FlowJo (Tree Star).

### Cytokine ELISAs

Cytokine levels were detected in culture supernatants and BALF by ELISA using monoclonal capture and biotinylated detection antibody pairs as follows, used at concentrations optimized previously: IL-4 [11B11 + BVD6-24G2 (BD Pharmingen)]; IL-13 [eBio13A + eBio1316H (eBioscience)]; IL-33 (R&D Duoset). *p*-nitrophenyl phosphate (pNPP, 1 mg/ml, Sigma) was used as a substrate. OD was measured at 405 nm on a Precision microplate reader (Molecular Devices) and data analyzed using Softmax Pro software.

### Antibody ELISAs

Serum antibodies to HES were measured by ELISA as previously described (7). Briefly, plates were coated with 1 μg/ml HES in carbonate buffer, blocked with 10% BSA in carbonate buffer, and incubated with serial dilutions of sera. Antibody binding was detected using HRP-conjugated goat anti-mouse IgA or IgG1 (Southern Biotech 1070–50 and 1040–50) and ABTS Peroxidase Substrate (KPL), and read at 405 nm.

### Gut Homogenate

Approximately 1 cm small intestine was homogenized in 500 μl 1x lysis buffer (Cell Signaling Technology Inc) plus 5 μl phenylmethanesulfonyl fluoride solution (PMSF) (Sigma) using a TissueLyser (Qiagen). Samples were centrifuged at 12,000 rpm for 10 min to remove debris and supernatants added to ELISAs, at a 1:10 dilution, to measure RELM-α (Peprotech) and Chil3 (R&D). Levels were normalized to total protein content measured using a Bradford assay. The same ELISA sets were used to analyse peritoneal lavage levels of RELM-α and Chil3.

### Immunohistochemistry

Transverse sections were made from 2 cm of paraffin-embedded small intestine, at a thickness of 4 μm using a cryostat. For MIF staining, sections were deparaffinized by immersing slides in Histoclear (Brunel Microscopes Ltd) for 5 min, and then hydrated through 100, 95, and 70% ethanol successively. Antigen retrieval was undertaken with citrate buffer (20 mM citric acid + 0.05% Tween 20 at pH6) warmed to 95°C for 20 min. Sections were blocked in 1x PBS with 1% BSA, 2% normal rabbit serum, 0.1% Triton X-100 and 0.05% Tween 20 for 30 min at room temperature and then incubated with rabbit α-MIF (Invitrogen) at 1:2000 dilution in block buffer, and left overnight at 4°C. Slides were immersed in 3% H_2_O_2_ for 10 min at room temperature, and washed in PBS. Goat α-rabbit conjugated to biotin (Vector Laboratories) at 5 μg/ml in PBS was added for 1 h at room temperature, in the dark. Following 2 washes in PBS, several drops of ABC Vectastain (Vector Laboratories) were added and slides left for 30 min at room temperature, in the dark. Slides were washed twice in PBS and DAB peroxidase solution (Vector Laboratories) was added for 5 min (until a brown stain had developed). With water washes in between, the following were added successively to counterstain the sections: Harris hemotoxylin solution (Sigma), acid alcohol (75% ethanol + 1% HCl) and Scott's Tap Water Substitute (ddH2O + 42 mM NaHCO3 and 167 mM MgSO4). Slides were dehydrated through 75, 95, and 100% ethanol and then Histoclear added for 5 min. Coverslips were added with DPX mountant (Sigma) and slides were left to dry overnight, in the dark. Pictures were taken using a Leica DFC290 compound microscope and Leica Application Suite software.

For fluorescent staining of macrophages, proximal small intestinal tissue was harvested and longitudinally opened. Any food matter was then gently removed by scraping and the tissue rolled onto a toothpick. Tissue was immediately immersed in OCT compound (Tissue-Tek), frozen on dry ice and stored at −80°C. Then, 13 μm thick sections were cut using a cryotome (Thermo Fisher), attached to positively charged microscope slides (VWR), dried for 15 min at room temperature and then stored at −80°C. For the staining procedure, tissue sections were thawed at room temperature and dried for 15 min under airflow, followed by fixation with 4% paraformaldehyde in PBS for 15 min at room temperature. The sections were then rinsed twice in PBS, permeabilised in PBS/0.1% saponin (Sigma-Aldrich) and consecutively blocked using a solution of 10% donkey serum (Abcam) and 0.3 M glycine (Fisher Scientific) in PBS for 60 min at room temperature. After 2 washes in PBS/0.1% saponin, primary staining was performed overnight at 4°C in PBS/1%BSA/0.1% saponin containing an antibody cocktail of: rat anti-mouse CD68-FITC (FA-11, Biolegend used at 5 ug/mL), rat anti-mouse EpCam-PE (G8.8, Biolegend, 2.5 ug/mL) and sheep anti-human/mouse arginase 1 (R&D, 5 ug/mL). Stained tissue sections were then washed 3 times in PBS/0.1% saponin and secondary antibody staining was performed with donkey anti-sheep AF647 (Abcam, 1/500) for 40 min at room temperature. This was followed by 3 washes in PBS/0.1% saponin and 2 PBS washes. DAPI containing Vectashield mounting media (Vector) and coverslips were applied prior to imaging using an EVOS FL Auto 2 fluorescence microscope (Invitrogen). Mean fluorescence intensity (MFI) of Arginase-1 staining in granulomas was analyzed using the image analysis software Fiji (SciJava).

### RNA Extraction and Quantitative PCR

To isolate mRNA from MLN and duodenal tissue, samples were first immersed in 1 ml of Trizol (Invitrogen) and disrupted using a TissueLyser (Qiagen) for 2 min at 25 Hz and then stored at −80°C until mRNA isolation was performed with the Qiagen mRNA easy kit (Qiagen) according to manufacturer's instructions. For duodenal analysis, ~0.5 cm of the uppermost part of the duodenum was sampled. Briefly, tissue was first disrupted using a TissueLyser (Qiagen), then 200 μl chloroform was added and samples were centrifuged at 12,000 g for 15 min at 4°C. The upper aqueous layer was recovered and added to 500 μl of isopropanol, mixed, and stood at room temperature for 10 min. The sample was then centrifuged again at 12,000 g for 10 min at 4°C. Pelleted RNA was washed once in 70% ethanol, and allowed to air dry before being dissolved in 50 μl of DEPC-treated water; 15 μl RNA was treated with DNAse (DNAFree kit, Ambion), concentrations were determined using a Nanodrop 1000 (Thermo Scientific), and samples reverse-transcribed using 1–2 μg of RNA with M-MLV reverse transcriptase (Promega). A PCR block (Peltier Thermal Cycler, MJ Research) was used for the transcription reaction at 37°C for 60 min. Gene transcript levels were measured by real-time PCR on a Roche Lightcycler 480 II, in 10 μl total volume made up of 4 μl cDNA, 5 μl SYBR Green (Roche), 0.3 μl of each primer (10 μM), and 0.4 μl DEPC treated water (Ambion) using standard conditions for 60 cycles. Target gene expression levels were normalized against the housekeeping gene GAPDH.

Primer sequences were as follows:

ARL2BP ADP-ribosylation factor-like binding protein
F: CGTATCCCAGGCTTCAACA
R: TGTGAGCAGCATGTCAAAGA
PHC2 Polyhomeotic 2
F: CCC ACA AAA TGG AAT GTA GAG G
R: ACT CCT CCG CGA TCT CCT.

### Array and Analysis

Two independent array experiments were conducted; in one, wild type BALB/c or MIF-deficient mice were infected with *H. polygyrus* for 5 days with tissues from the duodenum and MLN being collected and stored in RNA Later (Ambion) prior to processing. Duodenal tissues from uninfected BALB/c and MIF-deficient mice were also taken and stored (4 mice per group for each condition). MLNs from uninfected mice were too small to include in this experiment. In the second experiment, duodenal tissues were taken from naive mice (day 0) as well as days 3 and 7 of *H. polygyrus* infection for both BALB/c and MIF-deficient mice (4 mice per group for each condition).

Total RNA from tissues was extracted by firstly placing tissue in RLT buffer (Invitrogen) and then homogenized using a Tissue Lyser II (Qiagen) set for 2 min at 25 Hz. RNA isolation was performed with the RNeasy mini kit (Qiagen) according to manufacturer's instructions. RNA amplification and biotinylation prior to array hybridization was performed using the Illumina TotalPrep RNA Amplification kit (Ambion) according to manufacturer's instructions. All samples were checked for RNA quality prior to hybridization by Agilent 2100 Bioanalyzer (Agilent).

Data were generated at the Wellcome Trust Clinical Research Facility (WTCRF) located at Western General Hospital, Crewe Road South, Edinburgh, EH4 2XU. A total of 48 Illumina MouseWG6_V2_0_R3_11278593_A arrays were QC analyzed using the arrayQualityMetrics Bioconductor package to identify sub-standard and/or outlier arrays. Three arrays were identified as outliers and were removed from subsequent analyses.

### Software and Statistics

All statistical analyses were performed using Prism (Graphpad Software Inc.). Error bars on graphs display mean and standard error the mean (SEM). Student's *t*-test was used to compare groups. n.s., not significant, ^*^p < 0.05, ^**^p < 0.01, ^***^p < 0.001. Results are combined from several similar experiments unless otherwise stated in the figure legend.

## Results

### Abated Anti-helminth Immunity in the Absence of MIF

**Figure 1:**
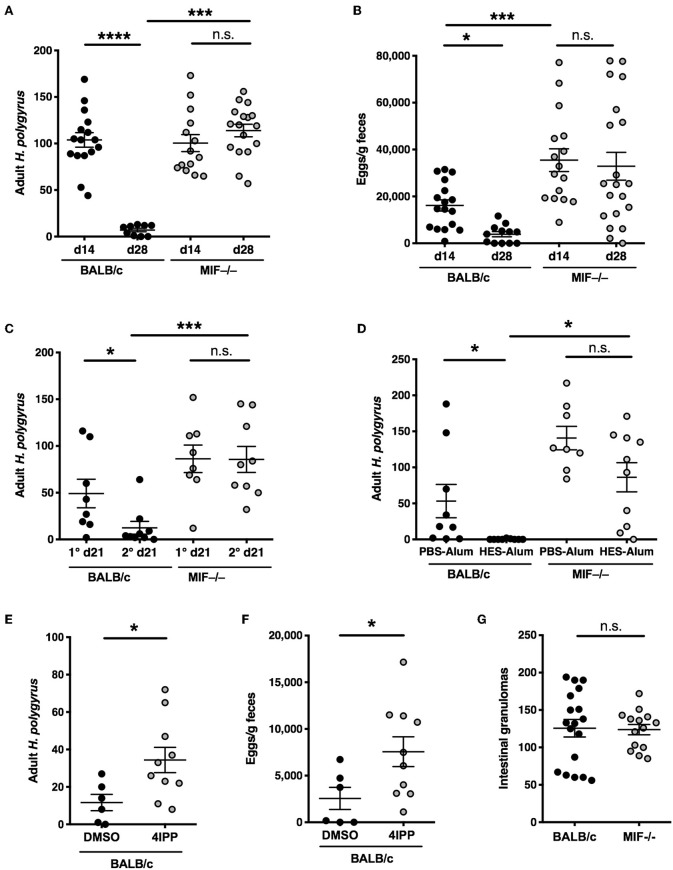
Compromised anti-helminth immunity in the absence of MIF. **(A,B)** Differential susceptibility of BALB/c and MIF^−/−^ mice to primary infection with *H. polygyrus*. Adult worm burdens in the small intestine **(A)** and fecal egg counts **(B)** were determined at days 14 and 28 post-infection with 200 *H. polygyrus* larvae by gavage. Data shown are combined from 3 independent experiments. **(C)** Differential induction of immunity following drug-abbreviated primary infection in BALB/c and MIF^−/−^ mice. At day 28 following infection with *H. polygyrus*, mice were given 2.5 mg of pyrantel embonate by oral gavage, twice over 24 h. After a further 14 days, mice were reinfected (or infected for the first time in 1° groups). Adult worms were enumerated at day 21 post infection. Data shown are combined from 2 independent experiments. **(D)** Differential expression of vaccine-induced immunity in BALB/c and MIF^−/−^ mice. At day 0, mice were injected with 10 μg of HES in alum, or PBS-alum control, followed by booster injections of 1 μg HES or PBS at days 28 and 35. On day 42 all mice were infected with *H. polygyrus*. Adult worm burdens were counted at day 21 post-infection. Data shown are combined from 2 independent experiments. **(E,F)** The MIF inhibitor 4-iodo-6-phenylpyrimidine (4-IPP) inhibits immunity in BALB/c mice infected with *H. polygyrus*. 1 mg 4-IPP in DMSO or DMSO alone was injected i.p. at days −1, 0, 2, 4, and 6 post-infection. Adult worms **(F)** and egg burdens **(G)** were enumerated at day 28 post-infection. Data shown are combined from 2 independent experiments. **(G)** Numbers of intestinal granulomas in BALB/c and MIF^−/−^ mice 14 days following primary infection with *H. polygyrus*. Data shown are combined from 2 independent experiments. n.s., not significant, *p < 0.05, ***p < 0.001, ****p < 0.0001.

A widely used model system for helminth infection is that of *H. polygyrus* in which parasitic larvae invade the intestinal tract and mature to luminal-dwelling adult worms releasing eggs into the environment (25, 26). BALB/c mice are initially susceptible to infection but are able to gradually reduce their worm load over several weeks through a macrophage-dependent mechanism (7), and are almost fully clear of adult worms (Figure 1A) and fecal eggs (Figure 1B) by day 28 post-infection. We tested MIF-deficient BALB/c mice and found that they were unable to reduce adult worm burdens or egg output following a primary *H. polygyrus* infection.

We then tested resistance of MIF^−/−^ mice to parasite infection in two models of acquired immunity. In the first, immunity to infection can be accelerated by a prior episode of abbreviated infection, terminated by anthelmintic therapy (27); in this case, alternatively-activated (M2) macrophages have been shown to be essential for protection (28). We found that MIF-deficient mice are unable to expel adult worms, which are mostly cleared by day 21 in the wild-type mice (Figure 1C). Secondly, we used a vaccine model in which sterilizing immunity is elicited by immunization with *H. polygyrus* excretory-secretory (ES) products in alum adjuvant (24). In this setting, BALB/c mice show complete protection but MIF-deficient animals fail to clear the parasites (Figure 1D).

We also reproduced the phenotype using a pharmacological inhibitor of MIF, 4-iodo-6-phenylpyrimidine (4-IPP), which acts as a “suicide substrate” by covalently binding the *N*-terminal proline required for catalytic activity (17, 22). Mice receiving this inhibitor showed significantly greater susceptibility than vehicle-treated mice to *H. polygyrus*, in terms of both adult worm burdens and egg output (Figures 1E,F).

Although immunity generally correlates with the formation of intestinal granulomas (29, 30), we found that MIF^−/−^ mice developed normal numbers of granulomas despite being completely susceptible to infection (Figure 1G).

### Intact Adaptive Type 2 Responses in MIF-Deficient Mice

**Figure 2:**
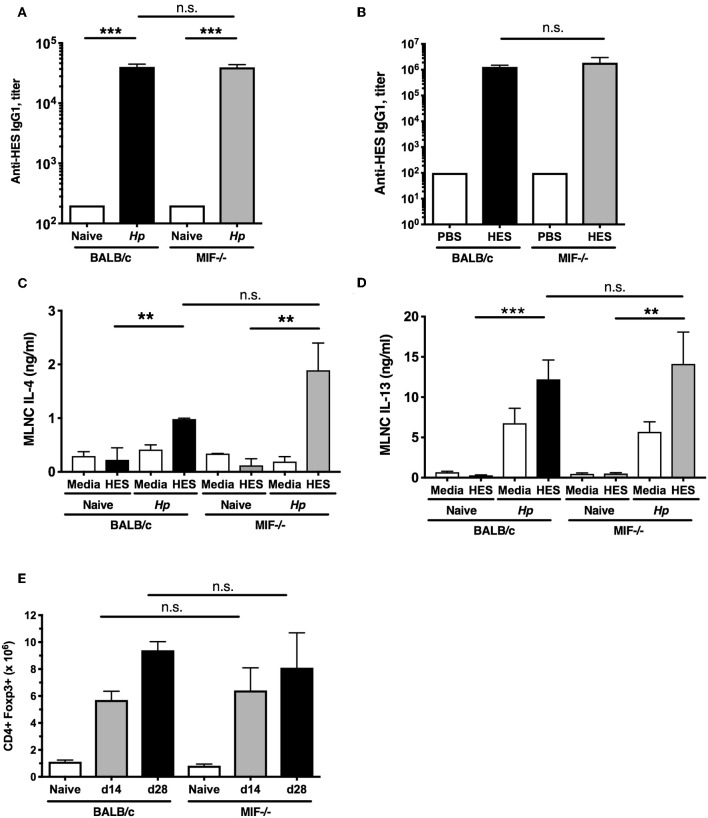
Intact adaptive and regulatory responses in MIF-deficient mice. **(A)** Comparable anti-helminth humoral immunity in BALB/c and MIF^−/−^ mice. Titers of HES-specific IgG1 serum antibodies from naïve and day 28-infected BALB/c and MIF^−/−^ mice, assessed by ELISA. Data are representative of two independent experiments. **(B)** Comparable anti-helminth humoral immunity in HES-vaccinated BALB/c and MIF^−/−^ mice. Parasite-specific antibody responses in vaccinated BALB/c and MIF^−/−^ mice. HES-specific serum IgG1 levels were measured by ELISA on the day of challenge infection. Data are representative of two independent experiments. **(C,D)** Comparable adaptive type 2 immune responses in BALB/c and MIF^−/−^ mice. Th2 cytokines from culture medium of MLNC from naïve and day 14 *H. polygyrus*-infected BALB/c and MIF^−/−^ mice, restimulated with 1 μg/ml HES or media for 72 hours. Levels of IL-4 **(C)** and IL-13 **(D)** were measured by ELISA. Data are representative of two independent experiments. **(E)** Regulatory cell induction by helminth infection is comparable between BALB/c and MIF^−/−^ mice. Numbers of CD4^+^Foxp3^+^ Treg cells within MLNs from BALB/c and MIF^−/−^ mice at days 14 and 28 post-infection with *H. polygyrus*. Data are representative of two independent experiments. n.s., not significant, **p < 0.01, ***p < 0.001.

Immunity to *H. polygyrus* following either drug-mediated clearance, or HES vaccination, has been shown to be antibody-dependent, in particular requiring IgG1 (24, 31). We therefore compared serological responses to infection in wild-type and MIF^−/−^ mice but found no difference in serum IgG1 titer (Figure 2A). In addition, both genotypes responded with equally high anti-HES antibody titers following vaccination (Figure 2B), although only the MIF^−/−^ animals failed to expel parasites. These findings implicated a deficiency in the cellular arm of the response in the absence of MIF.

We then compared parasite-specific T cell responses, as immunity to *H. polygyrus* is strongly Th2 dependent (32), by challenging small intestine draining mesenteric lymph node (MLN) cells from *H. polygyrus-*infected BALB/c and MIF^−/−^ mice with HES antigens. We found comparably robust IL-4 and IL-13 responses in both strains (Figures 2C,D); no induction of antigen-specific IFNγ responses above background was seen in either strain (data not shown), indicating the susceptibility of the MIF-deficient mouse cannot be explained by a switch to the Th1 mode of immunity.

Regulatory T cells (Treg) expressing the Foxp3^+^ transcription factor are known to expand during *H. polygyrus* infection (33, 34) and render mice susceptible (35, 36). MLN cell populations were analyzed by flow cytometry at 14 and 28 days post-infection, and similar increases in Foxp3^+^ Treg numbers were seen in both wild-type and MIF^−/−^ mice infected with *H. polygyrus* (Figure 2E). Increases in Treg frequency (as percentage of total CD4^+^ cells) and Foxp3 intensity were also similar between the two strains (data not shown), indicating that increased Treg activity is not contributing to greater susceptibility in the gene-targeted mice.

### Impaired Innate Type 2 Responses in MIF-Deficient Mice

**Figure 3:**
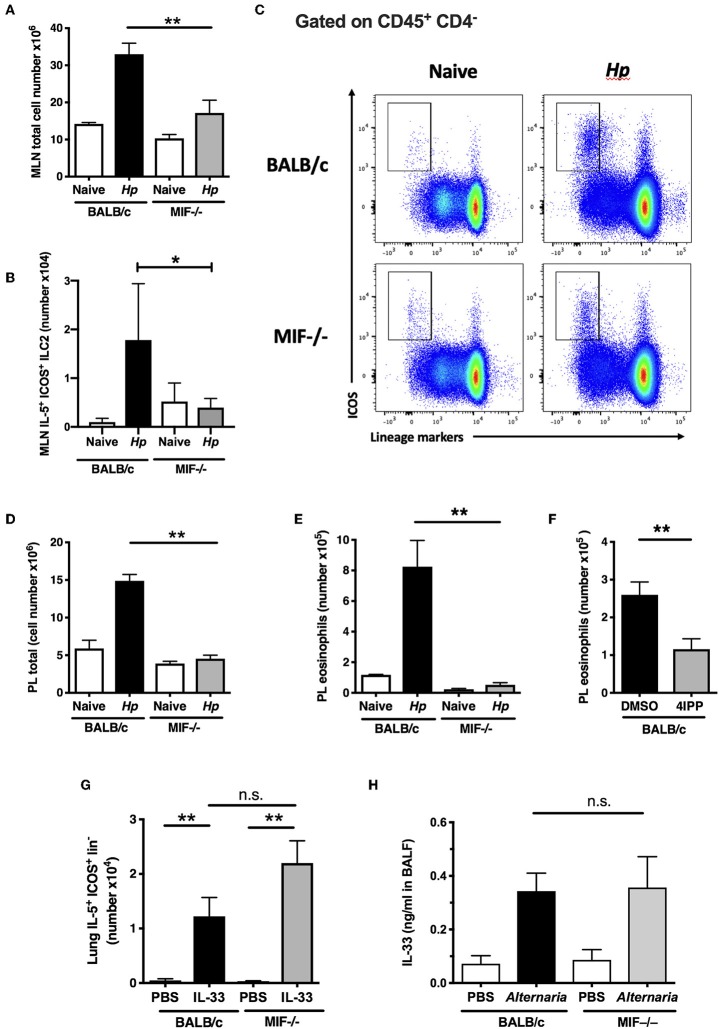
Impaired innate type 2 responses in MIF-deficient mice. **(A)** Total numbers of MLN cells recovered from BALB/c and MIF^−/−^ mice 7 days following *H. polygyrus* infection. Data are representative of two independent experiments. **(B)** Differential induction of ILC2s in BALB/c and MIF^−/−^ mice following *H. polygyrus* infection. Total numbers of IL-5^+^ ICOS^+^ lineage^−^ ILC2s within MLNC from naïve and d7 *H. polygyrus*-infected BALB/c and MIF^−/−^ mice. Data are representative of two independent experiments. **(C)** Representative flow cytometry plots of ICOS vs. Lineage markers in MLN cells from BALB/c and MIF^−/−^ naive mice and 7 days following *H. polygyrus* infection. **(D)** Total numbers of peritoneal lavage cells recovered from BALB/c and MIF^−/−^ mice 7 days following *H. polygyrus* infection. Data are representative of two independent experiments. **(E)** Differential induction of eosinophilia in BALB/c and MIF^−/−^ mice following *H. polygyrus* infection. Total numbers of eosinophils (SiglecF^+^ CD11b^+^) in naïve and *H. polygyrus-*infected BALB/c or MIF^−/−^ mice within the peritoneal lavage, at day 7 post-infection. Data are representative of two independent experiments. **(F)** The MIF inhibitor 4-IPP inhibits eosinophilia in BALB/c mice infected with *H. polygyrus*. Eosinophil numbers at day 7 post-infection with *H. polygyrus* in BALB/c following administration of 1 mg of the MIF inhibitor, 4-IPP, assessed as SiglecF^+^CD11b^+^ cells within the peritoneal lavage. Results are combined from two experiments with similar results. **(G)** MIF^−/−^ mice do not have an intrinsic defect in ILC2 induction. IL-5^+^ ILC2s as a proportion of live cells in digested lung tissue of BALB/c or MIF^−/−^ mice treated intranasally with PBS or rIL-33, measured by flow cytometry. Data are representative of two independent experiments, and were analyzed by nonparametric statistics. **(H)** MIF^−/−^ mice have normal ability to release the key alarmin IL-33 upon stimulation. Levels of IL-33 in BALF of BALB/c or MIF^−/−^ mice 1 h after intranasal administration of 10 μg *Alternaria* antigen, measured by ELISA. Results are combined from two experiments with similar results. n.s., not significant, *p < 0.05, **p < 0.01.

We then analyzed innate immune cell responses in BALB/c and MIF^−/−^ mice at day 7 post-*H. polygyrus* infection. In the wild-type animals, infection provokes a sharp increase in cell numbers within the MLNs (Figure 3A), which is diminished in the MIF^−/−^ mice. Infection also results in activation of ILC2s to express IL-5 which is almost totally ablated in the MIF-deficient animals (Figure 3B), within an overall reduction of ILCS in the MLN (Figure 3C). Likewise, cellular expansion in the peritoneal cavity provoked by infection is profoundly reduced in MIF-deficient mice (Figure 3D), as is eosinophilia (Figure 3E). The loss of eosinophils in the absence of MIF has previously been observed in both helminth infection and airway asthma models (13, 37, 38). We also tested the MIF-dependence of eosinophilia by administering the 4-IPP MIF inhibitor at the time of *H. polygyrus* infection. Wild-type mice receiving this inhibitor showed significantly reduced peritoneal eosinophilia compared to animals treated with the DMSO vehicle alone (Figure 3F).

To ascertain whether ILC2 differentiation was intrinsically compromised in MIF-deficient mice, we first tested the effects of exogenous IL-33 injection on the activation of ILCs in the lung; IL-33 drove equivalent IL-5^+^ ILC2 responses irrespective of MIF genotype (Figure 3G). We then tested the response of mice to airway challenge with *Alternaria* allergen, a potent stimulator of the ILC2 population through provoking rapid release of IL-33 from the airway epithelium (39). The introduction of exogenous *Alternaria* antigen elicited equivalent levels of IL-33 into the bronchoalveolar lavage after 1 h in BALB/c and MIF-deficient animals (Figure 3H), arguing that both the release of ILC2-stimulating alarmins and the development of ILC2 responses to these cytokines are intact in the MIF-deficient setting.

The diminished eosinophil responses in MIF-deficient mice cannot readily account for their increased susceptibility, as eosinophil-deficient ΔdblGATA mice retain their ability to expel *H. polygyrus* following vaccination (24). In addition, the poor ILC2 response observed did not translate into any shortfall in the Th2 response that develops to parasite antigens (Figures 2C,D), although it is possible that abated ILC2 production of IL-5 explains the deficient eosinophil responses in MIF^−/−^ mice.

### Type 2 Myeloid Responses in MIF-Deficient Mice

**Figure 4:**
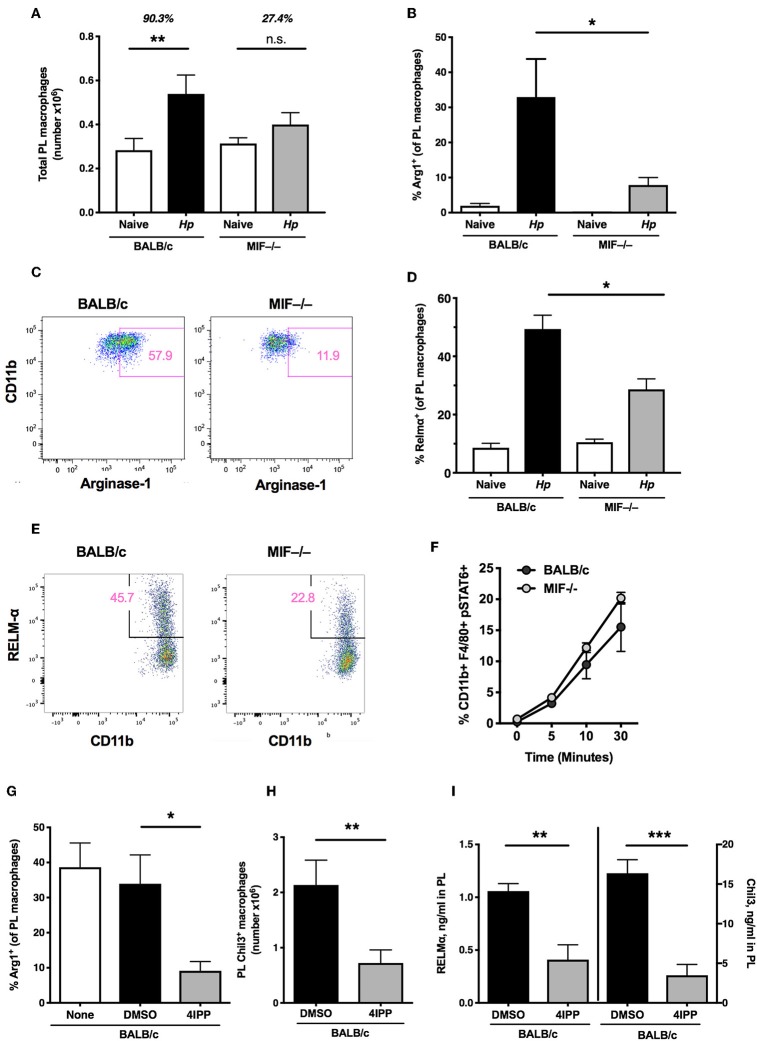
Type 2 myeloid responses in MIF-deficient mice. **(A)** MIF^−/−^ mice have a reduced capacity for induction of macrophages after helminth infection. Total macrophage (CD11b^+^F4/80^+^) numbers within the peritoneal cavity of BALB/c and MIF^−/−^ mice at day 6 post-infection with *H. polygyrus*, or in naïve mice. Data are representative of two independent experiments. **(B)** Percentage of peritoneal macrophages that are Arginase^+^ in BALB/c and MIF^−/−^ mice at day 3 post-infection with *H. polygyrus*, or in naïve mice. Results are combined from two experiments with similar results. **(C)** Representative flow cytometry plots of CD11b and Arginase-1 staining in peritoneal macrophages from BALB/c and MIF^−/−^ mice. **(D)** Percentage of peritoneal macrophages that are RELMα^+^ in BALB/c and MIF^−/−^ mice at day 6 post-infection with *H. polygyrus*, or in naïve mice. Data are representative of two independent experiments. **(E)** Representative flow cytometry plots of CD11b and RELM-α staining in peritoneal macrophages from BALB/c and MIF^−/−^ mice. **(F)** STAT6 phosphorylation of bone marrow-derived macrophages in response to 20 ng/ml recombinant IL-4, measured by flow cytometry at the times indicated. **(G)** The MIF inhibitor, 4IPP, can replicate the macrophage deficit of MIF^−/−^ mice in BALB/c mice after *H. polygyrus* infection. Percentage of peritoneal macrophages that are Arginase-1^+^ in DMSO- or 4IPP- treated BALB/c mice at day 3 post-infection with *H. polygyrus*, or in naïve mice receiving no treatment. Data are representative of two independent experiments. **(H)** Number of peritoneal macrophages that are Chil3^+^ in DMSO- or 4IPP-treated BALB/c mice at day 7 post-infection with *H. polygyrus*. Results are combined from two experiments with similar results. **(I)** Levels of RELMα and Chil3 measured by ELISA in peritoneal lavage fluid of DMSO- or 4IPP- treated BALB/c mice at day 7 post-infection with *H. polygyrus*. Data are representative of two independent experiments. *p< 0.05, **p < 0.01, ***p < 0.001.

We next analyzed myeloid subpopulations, which play critical roles in mediating immunity in many helminth settings (7, 28, 40). To establish whether the absence of MIF resulted in significant differences within the myeloid compartment, we compared the phenotype of CD11b^+^ F4/80^+^ macrophages in MIF-sufficient and -deficient mice. We found that, following *H. polygyrus* infection, few viable lamina propria cells could be recovered from either BALB/c or MIF-deficient mice and hence populations were assayed from the peritoneal cavity, in which there is extensive expansion and alternative activation of macrophages during the first week of infection (41). Notably, the increase in macrophage numbers was muted in the peritoneal cavity of MIF-deficient mice (27% above naïve levels) compared to wild-type animals (90% increased) (Figure 4A).

Because MIF has previously been shown to promote the alternative activation of macrophages, alongside IL-4Rα-binding cytokines (16), we measured expression of key alternatively-activated macrophage (AAM)-associated gene products Arginase-1, Chil3 (Ym1) and RELMα by a combination of flow cytometry of peritoneal cell populations, and ELISA for soluble proteins in peritoneal lavage fluids. By each of these measures MIF^−/−^ mice showed significant impairment of alternative activation. Thus, the proportions of peritoneal macrophages staining for Arginase-1 (Figures 4B,C) and RELMα (Figures 4D,E) were significantly reduced in MIF-deficient animals, as were levels of detectable Chil3 and RELMα protein in the lavage following *H. polygyrus* infection (see below). We also compared the level of IL-4-stimulated STAT6 phosphorylation in bone marrow-derived macrophages from MIF-sufficient and deficient mice, and found no significant differences (Figure 4F), indicating that the requirement for MIF acts downstream, or independently of, IL-4 receptor ligation.

We next examined the *in vivo* effects of pharmacological MIF inhibition on the expression of AAM markers; administration of 4-IPP significantly reduced the number of CD11b^+^ Arginase-1^+^ (Figure 4G) and Chil3^+^ (Figure 4H) peritoneal macrophages after *H. polygyrus* infection, as well as the levels of both Chil3 and RELMα protein in the peritoneal lavage fluid of BALB/c mice (Figure 4I).

**Figure 5:**
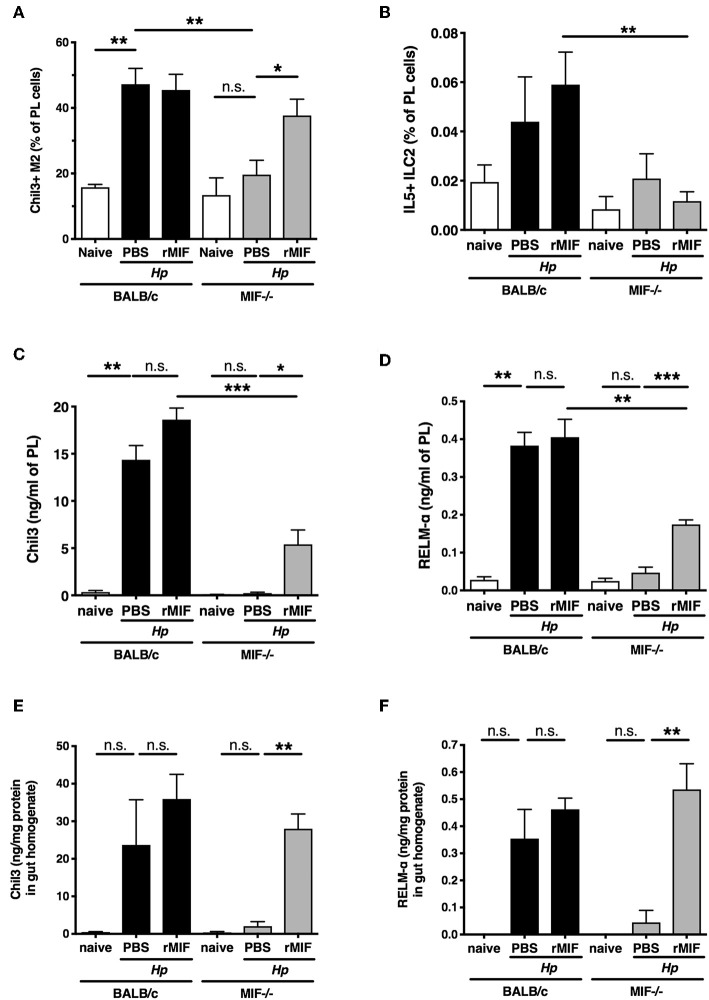
rMIF rescues the macrophage phenotype of MIF-deficient mice. **(A)** Administration of recombinant MIF can rescue the MIF^−/−^ phenotype after *H. polygyrus* infection. Percentage of peritoneal macrophages (CD11b^+^ F4/80^+^) that are Chil3^+^ in BALB/c or MIF^−/−^ mice treated with PBS or 50 μg recombinant MIF intraperitoneally, at day 7 post-infection with *H. polygyrus*, or in naïve mice. Results are combined from two experiments with similar results. **(B)** Percentage of PL cells that are IL-5^+^ ILC2s from the same experiments as **(A)**. Results are combined from two experiments with similar results. **(C,D)** Expression of Chil3 **(C)** and RELMα **(D)** in peritoneal lavage fluid, measured by ELISA, from the same experiments as **(A)**. Results are combined from two experiments with similar results. **(E,F)** Expression of Chil3 **(E)** and RELMα **(F)** in small intestinal homogenate, measured by ELISA, from the same experiments as **(A)**. Results are combined from two experiments with similar results. n.s., not significant, *p < 0.05, **p < 0.01, ***p < 0.001.

To test whether MIF is directly responsible for the alternative activation of macrophages, we evaluated the effects of administering recombinant MIF into the peritoneal cavity of MIF-deficient mice. Such treatment restored the proportions of Chil3^+^ AAM in this site after *H. polygyrus* infection to levels comparable with wild-type mice (Figure 5A), but did not rescue the significant deficit in ILC2 cells in the same location (Figure 5B). Exogenous MIF was able to partially restore protein levels of Chil3 and RELM-α in the peritoneal lavage fluid of MIF^−/−^ mice (Figures 5C,D), although remaining significantly below those of the wild-type mice, and no eosinophilia was elicited (data not shown). Furthermore, these products were also upregulated in small intestinal tissues of *H. polygyrus*-infected MIF-deficient mice (Figures 5E,F). As intraperitoneal delivery of MIF did not restore resistance to the parasite infection (data not shown), it is likely that localized production and release within the intestinal tract may be required for effective recruitment and activation of tissue macrophages at the site of infection.

**Figure 6:**
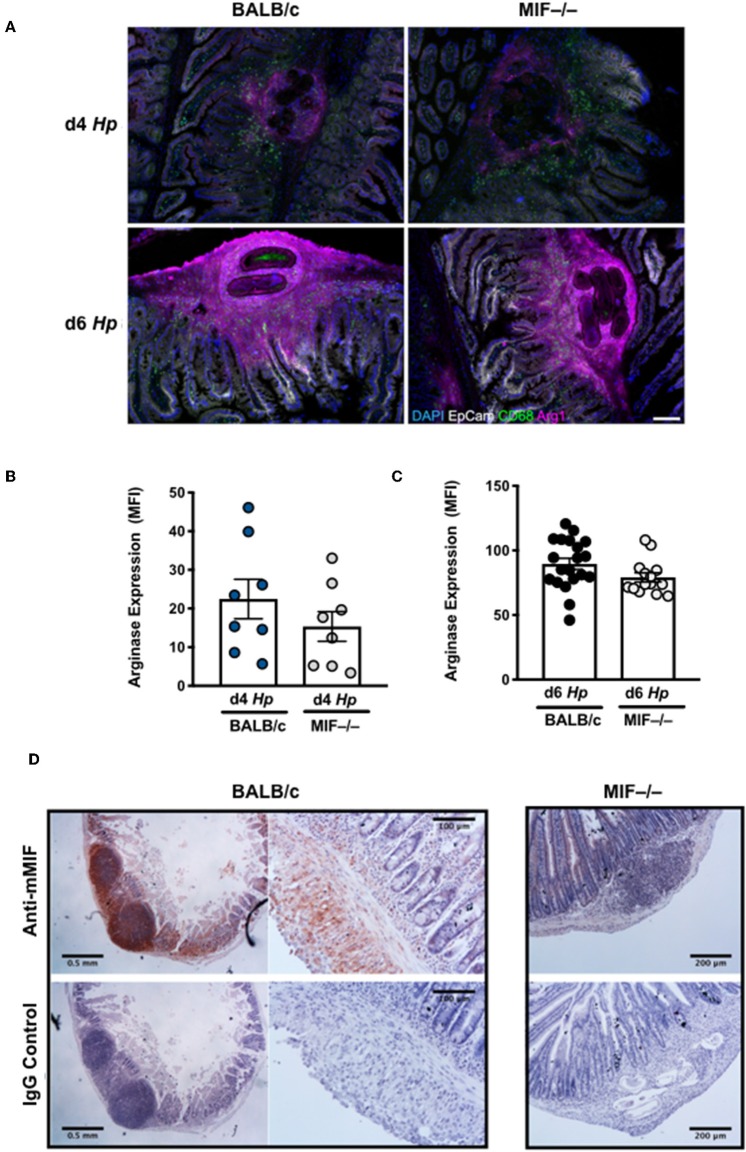
Myeloid cell infiltration around tissue larvae of *H. polygyrus* with expression of Arginase-1 and MIF. **(A)** M2 macrophages infiltrate the infection site of *H. polygyrus* larvae in the small intestine. Arginase, CD68 and EpCam staining of granulomas around larval parasites in the small intestinal submucosa at days 4 and 6 of *H. polygyrus* infection in BALB/c and MIF^−/−^ mice. Images are representative of two experiments with similar results. **(B,C)** Analysis of intensity of Arginase staining in granulomas at days 4 and 6 after *H. polygyrus* infection. For each group at each time point, granulomas were analyzed from 4 individual animals, and intensity data pooled. Each data point represents an individual granuloma. **(D)** MIF is expressed in the infection site of *H. polygyrus* larvae in the small intestine. Intense staining of polyclonal rabbit anti-MIF antibody is observed in the granulomas around larval parasites in the small intestinal submucosa at day 6 of *H. polygyrus* infection in BALB/c mice, but not in MIF^−/−^ animals or in sections stained with isotype control IgG. In addition, widespread specific antibody staining is seen throughout the submucosal tissue. Images were collected on a Leica compound microscope. Scale bars represent 500 and 250 μm.

While peritoneal macrophages may mirror the phenotype of the intestinal population, it is important to also study those cells closely associated with larval parasites in the submucosa of the small intestine, where *H. polygyrus* is found for the first 8 days of infection. We used immunofluorescence imaging to characterize the patterns of macrophage activation and accumulation around larval parasites, and their expression of Arginase-1 which is known to be required for immunity to this helminth (28). Surprisingly, local macrophage infiltration and overall Arginase-1 expression did not significantly differ in infected MIF^−/−^ mice (Figure 6A), and although the intensity of Arginase-1 staining in gene-deficient tissues was marginally weaker at day 4 of infection (Figure 6B), by day 6 it was as ubiquitous as in the wild-type controls (Figure 6C). In both examples, Arginase-1 is disseminated throughout the granuloma, indicating that it is either or both expressed by cells other than macrophages, and/or released extensively into the extracellular milieu from those cells which express it. Immunohistochemical staining was also used to identify widespread expression of MIF in intestinal tissues *in vivo*; in particular, MIF was intensely expressed within the granulomas centered around immobile larvae (Figure 6D), at the foci of the local immune response to intestinal helminth infection.

#### Gene Expression in *H. polygyrus*-Infected MIF-Deficient Mice

**Figure 7:**
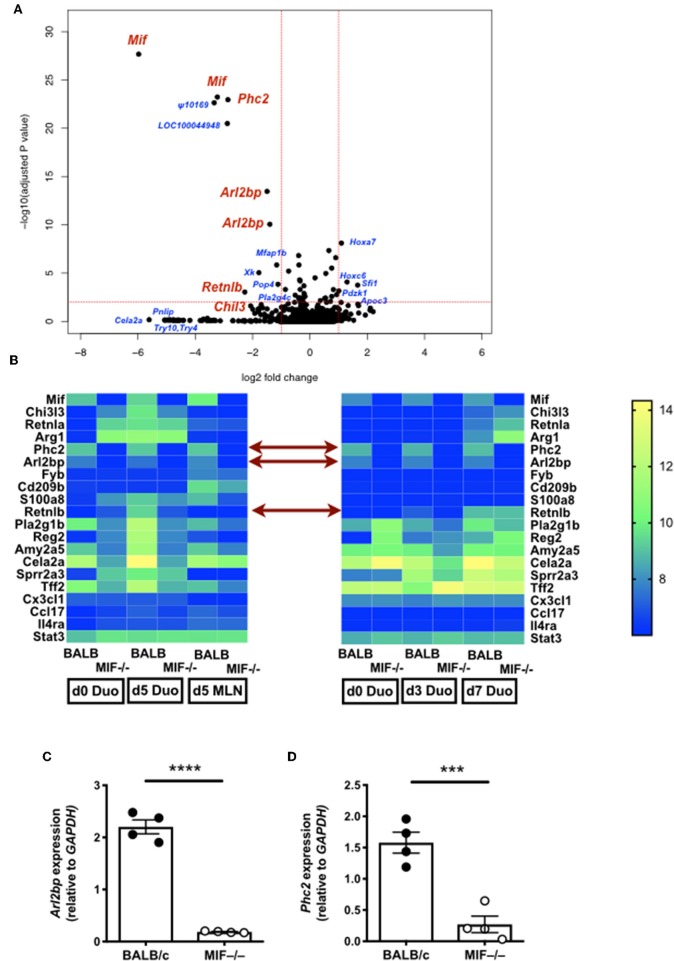
Gene expression in *H. polygyrus*-infected MIF-deficient mice. **(A)** Volcano plot comparing gene expression in duodenal tissue from BALB/c and MIF^−/−^ mice 5 days after *H. polygyrus* infection. Genes of interest are labeled in red; other loci showing large and/or significant changes are labeled in blue. **(B)** Heat maps from two gene expression experiments, on duodenal and MLN tissue for expression of selected genes 5 days after *H. polygyrus* infection in BALB/c and MIF^−/−^ mice (left hand panel), and of duodenal tissues at days 3 and 5 post-infection in the same genotype mice (right hand panel). Expression levels are colored from blue (lowest) to yellow(highest) and in each case represent the mean of 4 replicates. **(C,D)** RT-PCR validation of *Arl2bp* **(C)** and *Phc2* **(D)**. MLNs were harvested for analysis at d 5 of *H. polygyrus* infection, and subject to RT-PCR using gene specific primers. ***p < 0.001, ****p < 0.0001.

To gain insight into possible signaling and effector molecules dependent upon MIF in helminth infection, we compared gene expression profiles of MIF-sufficient and –deficient mice by array analyses of duodenal tissue taken 3, 5, and 7 days following *H. polygyrus* infection, as well as MLN sampled on day 5. As shown in Figures 7A,B, relatively few genes showed major expression changes but among them were *Arl2bp*, a little-studied gene encoding a protein which stabilizes nuclear localization of the STAT3 transcription factor (42), and *Phc2*, a central component of the Polycomb 1 complex that maintains epigenetic imprinting (43). In addition, a number of other genes showed either smaller or more transient reductions in levels in *H. polygyrus*-infected MIF^−/−^ mice compared to wild-type, including *Retnlb* (encoding RELM-β) and *Pla2g1b*, two epithelial-expressed genes reported to possess direct anti-helminth properties (44, 45).

#### STAT3 Signaling Contributes to Immunity to *H. polygyrus*

**Figure 8:**
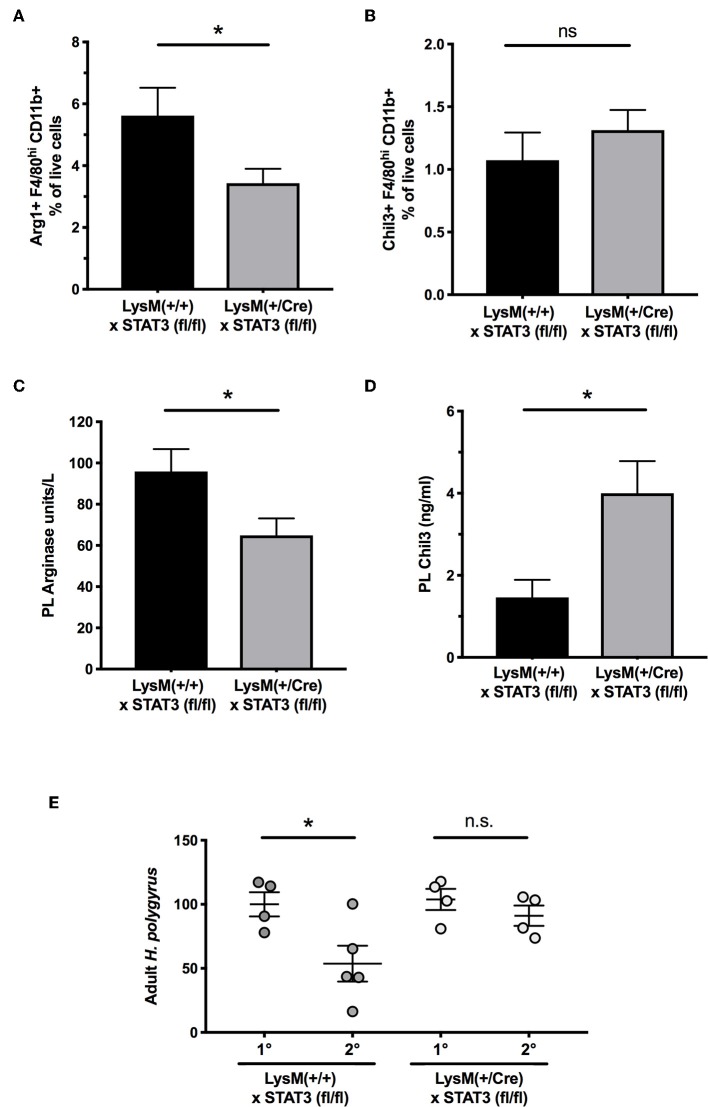
Myeloid cell expression of STAT3 is required for optimal Arginase-1 expression and for secondary immunity to *H polygyrus*. **(A)** Peritoneal macrophages were recovered from control and conditional STAT3 gene deleted mice, with genotypes of STAT3^fl/fl^xLysM-Cre^−/−^ and STAT3^fl/fl^xLysM-Cre^+/−^, respectively, and stained for surface CD11b and F4/80, together with intracellular Arginase-1. Staining was evaluated by flow cytometry. Data are pooled from three independent experiments. **(B)** Expression of Chil3 within the same macrophage populations. Data are pooled from three independent experiments. **(C)** Peritoneal lavage fluids were recovered from the same animals as in **(A,B)**, and functional soluble arginase levels measured by an enzyme assay *p < 0.05. **(D)** Presence of Chil3 protein in the same lavage fluids as **(C)** *p < 0.05. **(E)** Myeloid cell-specific deletion of STAT3 impairs secondary immunity. LysM^Cre^xSTAT3^fl/fl^ mice and STAT3^fl/fl^ controls) were infected with *H. polygyrus* and infections cleared with pyrantel embonate. Mice were subsequently challenged with a secondary infection, or infected for the first time for primary controls, and adult worms counted in the small intestine at d21 post infection. Results are combined from two experiments with similar results. n.s., not significant, *p < 0.05.

Gene expression differences for *Arl2bp* and *Phc2* were confirmed by RT-PCR on MLN samples at day 5 of infection of wild-type and MIF-deficient mice (Figures 7C,D). As abated *Arl2bp* expression may compromise STAT3 signaling, we then tested mice in which STAT3 expression had been blocked in myeloid lineages through transgenic expression of Cre recombinase under the LysM promoter, combined with homozygous alleles for a flox-flanked STAT3 (46). We first examined CD11b^+^F4/80^+^ peritoneal macrophages from *H. polygyrus*-infected mice, and found significantly fewer cells expressed intracellular Arginase-1 in mice carrying the myeloid-restricted deletion (Figure 8A); notably, Chil3+ cell numbers were similar, albeit low, in both genotypes (Figure 8B). We also collected peritoneal lavage fluid, and found significantly lower Arginase enzymatic activity in the STAT3-conditionally deleted mice (Figure 8C). In addition, we measured soluble Chil3 in the lavage fluid, which rather than being inhibited in mice lacking myeloid cell STAT3 expression, actually showed a significant increase (Figure 8D).

As the C57BL/6 background is fully susceptible to primary *H. polygyrus* infection, we then evaluated immunity to a secondary challenge with *H. polygyrus* following chemotherapeutic clearance of the primary infection. In wild-type mice lacking the Cre allele, there was significant protection against challenge, but in myeloid-specific STAT3-deleted animals, parasite loads were similar in primary and secondary infection, showing a failure of protective immunity in the STAT3-deficient setting (Figure 8E).

## Discussion

The crucial role of innate immune cell populations in immunity to helminths is well recognized (5, 47), but the molecular mediators required for their activation have not all been identified. Here we report that MIF is a critical cytokine required for clearance of the intestinal parasite *H. polygyrus*, impacting on multiple type 2 innate cell populations while not significantly affecting adaptive B or T cell responses. Although previously viewed as a pro-inflammatory agent in settings of sepsis and microbial challenge (48), our work and that of others demonstrate that its role is context-dependent, so that in the presence of the pivotal type 2 cytokine IL-4, MIF will synergize to induce characteristic M2 products including Chil3, RELMα and Arginase-1 (16, 17). Significantly, the activity of MIF is not confined to the macrophage lineage, with evident lesions in ILC numbers, and a profound loss of eosinophils, in MIF-deficient animals. These multiple facets of MIF are characteristic of a protein with a range of diverse activities that are remarkable for a protein of only 114 amino acids, and one discovered at the dawn of the cytokine era (49, 50). For example, MIF is also a non-consensus ligand of chemokine receptors (51), an inhibitor of intracellular signaling and inflammasome assembly (52, 53) and a partner in a nuclear DNA-cleaving complex (54).

Immunity to *H. polygyrus* is known to require a potent type 2 response, dependent upon CD4^+^ Th2 cells driving a specific IgG1 antibody response together with alternatively-activated (M2) macrophages stimulated through the IL-4R (7, 28, 55). Immunity can act in two distinct phases: firstly against tissue-dwelling larvae which are immobilized and killed in the setting of a challenge infection or an immunized host, and secondly against luminal adults which are cleared by the combined action of activated myeloid and epithelial cells (26). Importantly, parasites surviving immune attack in the tissues can emerge into the lumen with diminished fitness, resulting in lower egg production and shorter survival times. MIF deficiency was found not only to compromise worm expulsion in both naive and vaccinated animals, but also to result in significantly higher egg production at day 14 (Figure 1B), confirming that early responses to the tissue larvae are abated in the absence of MIF.

Among other innate cell populations, ILC2s can promote the response, but are not sufficient for expulsion (56), while eosinophils act to restrain the intestinal Th2 response to *H. polygyrus* (57) and eosinophil-deficient mice clear parasites promptly following immunization with a secreted antigen vaccine (24). As MIF-deficient mice mounted a normal adaptive B- and T cell response to infection, we concluded that these mice must lack a key innate effector population, which we propose are the IL-4R-dependent M2 macrophages. Indeed, even in the presence of memory Th2 cells known to drive M2-dependent immunity to *H. polygyrus* (28) immunity fails in the absence of MIF, again suggesting lesion(s) in the macrophage compartment. If so, this would argue that IL-4R ligation may require supplementation through other signals to achieve the fully activated M2 state required for worm expulsion. A further interesting point is that MIF-deficient mice generated a similar granuloma response to wild-type animals, and yet could not trap parasites even following vaccination. As cell recruitment to granulomas was similar in the two genotypes, and as MIF itself is highly expressed in the WT granuloma, it may be that MIF acts not at the level of differential cell recruitment, but by activating cells locally to promote immunity.

It is known that numbers of macrophages in *H. polygyrus* infection rapidly expand, notably in the peritoneal cavity, and adopt the M2 phenotype characterized by production of Arginase-1, Chil3 and RELMα (41). We noted a significant delay in peritoneal macrophage activation in MIF-deficient animals, with a lag also evident in production of these archetypal markers, although the effect was less obvious at the tissue site of infection. However, it is known that genetically resistant strains of mice mount a more rapid response to *H. polygyrus* (7), suggesting that retardation of M2 activation in the absence of MIF may account for failure of immunity. We further established the role of MIF in testing a pharmacological inhibitor, which recapitulated both impaired expression of M2 gene products, and greater susceptibility to infection, that are observed in the gene-targeted mice. In addition, we established exogenous MIF could restore the activation of peritoneal macrophages to the wild-type profile.

Previous work has demonstrated that immunity to *H. polygyrus* is compromised by clodronate depletion of macrophages (7), and by pharmacological blockade of Arginase-1, a principal product of M2 macrophages (28). However, we have yet to establish whether such macrophages activated by MIF would be sufficient to confer immunity by adoptive transfer to naive recipients. One obstacle to such an experiment is that appropriate migration of the adoptively transferred population to the site of infection may not occur, particularly given that MIF is thought to arrest macrophage migration *in situ*. We note, however, that in a related parasite model of *Nippostrongylus brasiliensis*, in which larvae transit the lung intranasal transfer of M2 macrophages significantly augments anti-parasite immunity (58).

To further analyse the role of MIF *in vivo*, we next compared gene expression profiles, in the intestinal tract and MLN. In these tissues, disparities in M2 macrophage products were not so apparent, but two transcripts markedly under-represented in the MIF-deficient state were *Arl2bp* and *Phc2*. The former is a STAT3 nuclear retention factor, raising the possibility that STAT3 is required for functional macrophage activity in *H. polygyrus* infection. While we did not observe any difference in STAT3 phosphorylation following IL-6 or IL-10 stimulation of wild-type or MIF-deficient cells (data not shown), *Arl2bp* may manifest a more subtle effect on nuclear activity which we could not detect. However, it has previously been shown that Arginase-1 and Chil3 expression are STAT-3 dependent in mammary epithelial cells (59), as is also the case for Arginase-1 in myeloid-derived suppressor cells (MDSCs) (60), which share some characteristics with M2 macrophages. Although global STAT3 deletion is lethal in mice, we were able to test animals with a myeloid-specific conditional deletion of STAT3, which show modest reduction in Arginase-1 expression during *H. polygyrus* infection and lose the ability to expel parasites on secondary exposure. As pharmacological inhibition of Arginase is also able to block expulsion of this parasite (28), these data may indicate that small changes in the timing or peak of Arginase production are sufficient to alter the outcome of infection. In addition, our finding that Chil3 is actually increased in myeloid-specific STAT3-deficient mice which fail to expel, would argue that despite its abundance, Chil3 is not a primary factor that promotes helminth clearance.

As mentioned above, MDSCs commonly express Arginase. They are also expanded *in vitro* by MIF (17, 61, 62), and their development is in part STAT3-dependent (63). Furthermore, MDSC transfer alone has been reported to hasten expulsion of the nematode species *N. brasiliensis* from mice (64). In addition, key enzymes such as amylases (*Amy2a5*, Figure 7B) which show ablated gene expression in MIF-deficient mice are reported to be up-regulated in tumor-associated MDSCs (65). However, MDSCs are a highly heterogenous grouping of myeloid cells (66), and further definition of which, if any, subset of these cells may play a role in helminth immunity will be an important future goal.

**Figure 9:**
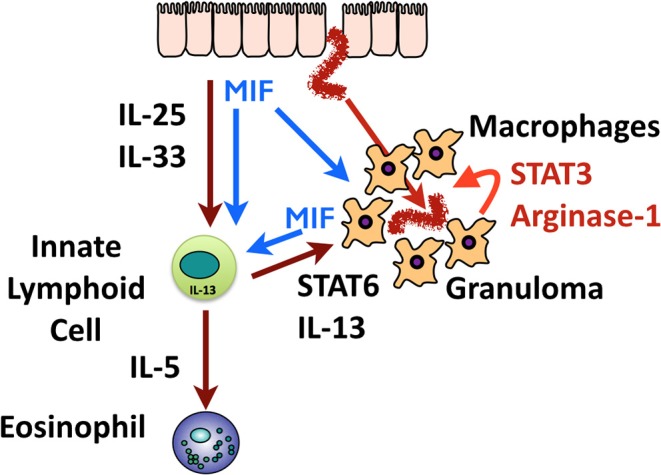
Graphical summary of the role of MIF in immunity to *H polygyrus*. Invading helminths elicit release of epithelial alarmins (IL-25 and IL-33) and most likely, MIF. These innate activators act on macrophages and ILCs; macrophages activated by MIF, and by IL-13 from ILCs, swarm around the tissue helminth to form the granuloma. High levels of MIF are also produced within the granuloma which may auto-stimulate macrophages, drive Arginase-1 expression, and act on distant ILCs. ILC2s also produce IL-5 which stimulates eosinophilia in the tissues.

We summarize the interactions discussed in this paper in Figure 9. While our data argue that in addition to IL-4R signaling, MIF, and STAT3, are each involved in macrophage immune function, we cannot exclude that other cell types respond to these signals, and are integrally required for parasite expulsion. For example, neutrophils play a role particularly in primary infection, as depletion of Gr1^+^ cells compromises primary immunity to *H. polygyrus* (24, 67) and can prime macrophages for resistance to challenge infection with this parasite (68).

Finally, in mice lacking MIF there was a substantial reduction of a polycomb 1 complex gene, *Phc2*, which we are now exploring. The polycomb complex mediates chromosomal imprinting (43) which is a central feature of macrophage commitment and innate immune memory (69–71). While *Phc2* itself has yet to be implicated in macrophage differentiation, other polycomb components are known to be involved (72), and epigenetic modifications have been found to be essential to the phenotypes of both M1 (73, 74) and M2 (75, 76) macrophages. Hence, there may be a longer-term inability of macrophages to fully polarize and form innate memory in the absence of MIF, which in turn could explain the failure to expel parasites in vaccinated animals.

With this report, MIF may be seen as joining the ranks of intestinal epithelial-derived mediators that recruit and sustain innate immune responses. However, MIF is produced and functions in many niches, and many critical features underpinning the source, stimulus and regulation of its expression remain to be determined. The discovery that additional stimuli are required for optimal alternative activation will also be important in defining the pathway through which the M2 phenotype is controlled. Indeed, a number of co-activating pathways for M2 macrophages have very recently been described including surfactant protein A (77) and markers of apoptosis (78) – as with MIF these ligands may prove indispensable in designing future interventions to generate protective immunity to the range of parasitic organisms for which type 2 immunity is critical.

## Data Availability Statement

Gene expression data have been deposited with the accession number GSE139009 and are available at https://www.ncbi.nlm.nih.gov/geo/query/acc.cgi?acc=GSE139009.

## Ethics Statement

This study was carried out in accordance with the policies of the University of Glasgow and the UK Home Office. The protocols were approved by the University of Glasgow Ethical Review Board.

## Author Contributions

KF designed and undertook the majority of experiments. FV, YH, JH, DS, SN, and SL undertook experiments. AI provided guidance on the design of and analyzed the RNA array experiment. SN and MR undertook the STAT3 flox experiments. MR and HM provided guidance and expertise. SL critically reviewed the manuscript. RM oversaw all work and wrote the paper.

### Conflict of Interest

The authors declare that the research was conducted in the absence of any commercial or financial relationships that could be construed as a potential conflict of interest.
